# Beyond-1000 nm low-energy sunlight-driven photocatalysis enabled by quantum dot-based photon upconversion

**DOI:** 10.1093/nsr/nwag078

**Published:** 2026-02-05

**Authors:** Lin-Han Jiang, Ming-Yu Zhang, Jia-Yao Li, Ran Li, Ying-Ze Li, Hong-Juan Feng, Weijian Sun, Xiaofei Miao, Junxue Liu, Wenbo Hu, Ling Huang, Dai-Wen Pang

**Affiliations:** Frontiers Science Center for New Organic Matter, Research Center for Analytical Sciences, College of Chemistry, State Key Laboratory of Medicinal Chemical Biology, Tianjin Key Laboratory of Biosensing and Molecular Recognition, Frontiers Science Center for Cell Responses, Haihe Laboratory of Sustainable Chemical Transformations, Nankai University, Tianjin 300071, China; Frontiers Science Center for New Organic Matter, Research Center for Analytical Sciences, College of Chemistry, State Key Laboratory of Medicinal Chemical Biology, Tianjin Key Laboratory of Biosensing and Molecular Recognition, Frontiers Science Center for Cell Responses, Haihe Laboratory of Sustainable Chemical Transformations, Nankai University, Tianjin 300071, China; Frontiers Science Center for New Organic Matter, Research Center for Analytical Sciences, College of Chemistry, State Key Laboratory of Medicinal Chemical Biology, Tianjin Key Laboratory of Biosensing and Molecular Recognition, Frontiers Science Center for Cell Responses, Haihe Laboratory of Sustainable Chemical Transformations, Nankai University, Tianjin 300071, China; Frontiers Science Center for New Organic Matter, Research Center for Analytical Sciences, College of Chemistry, State Key Laboratory of Medicinal Chemical Biology, Tianjin Key Laboratory of Biosensing and Molecular Recognition, Frontiers Science Center for Cell Responses, Haihe Laboratory of Sustainable Chemical Transformations, Nankai University, Tianjin 300071, China; Frontiers Science Center for New Organic Matter, Research Center for Analytical Sciences, College of Chemistry, State Key Laboratory of Medicinal Chemical Biology, Tianjin Key Laboratory of Biosensing and Molecular Recognition, Frontiers Science Center for Cell Responses, Haihe Laboratory of Sustainable Chemical Transformations, Nankai University, Tianjin 300071, China; Frontiers Science Center for New Organic Matter, Research Center for Analytical Sciences, College of Chemistry, State Key Laboratory of Medicinal Chemical Biology, Tianjin Key Laboratory of Biosensing and Molecular Recognition, Frontiers Science Center for Cell Responses, Haihe Laboratory of Sustainable Chemical Transformations, Nankai University, Tianjin 300071, China; Time-Tech Spectra Co., Ltd., Dalian 116085, China; Frontiers Science Center for Flexible Electronics and Xi’an Institute of Flexible Electronics (IFE), Northwestern Polytechnical University, Xi’an 710072, China; Time-Tech Spectra Co., Ltd., Dalian 116085, China; Frontiers Science Center for Flexible Electronics and Xi’an Institute of Flexible Electronics (IFE), Northwestern Polytechnical University, Xi’an 710072, China; Frontiers Science Center for New Organic Matter, Research Center for Analytical Sciences, College of Chemistry, State Key Laboratory of Medicinal Chemical Biology, Tianjin Key Laboratory of Biosensing and Molecular Recognition, Frontiers Science Center for Cell Responses, Haihe Laboratory of Sustainable Chemical Transformations, Nankai University, Tianjin 300071, China; Frontiers Science Center for New Organic Matter, Research Center for Analytical Sciences, College of Chemistry, State Key Laboratory of Medicinal Chemical Biology, Tianjin Key Laboratory of Biosensing and Molecular Recognition, Frontiers Science Center for Cell Responses, Haihe Laboratory of Sustainable Chemical Transformations, Nankai University, Tianjin 300071, China

**Keywords:** solar energy, photochemistry, NIR-II photon upconversion, quantum dots, triplet exciton transfer

## Abstract

Solar energy harvesting and conversion are pivotal to sustainable chemistry and green chemistry, yet fundamental bottlenecks persist. A key unresolved challenge is chemical transformations driven by low-energy sunlight, especially beyond 1000 nm, which is limited by insufficient absorption and low photon energy. Here, we employ PbS quantum dots (QDs) as near-infrared-II (NIR-II) absorbers and precisely modulate the CdS shell to balance the triplet exciton transfer efficiency with the triplet lifetime of the surface ligands, thereby enhancing the overall sensitization performance of the hybrid photosensitizer. Coupled with rubrene as the annihilator, a record upconversion efficiency of 3.9% was achieved under 1064 nm excitation. Furthermore, the efficient upconversion material enables unprecedented beyond-1000 nm low-energy sunlight-driven large-volume photocatalysis, applicable to both free radical polymerization and atom transfer radical polymerization. This work establishes a foundation for advanced solar energy technologies with broad implications for photocatalysis and next-generation photovoltaics.

## Teaser

By optimizing the CdS shell thickness to modulate triplet exciton transfer, a record NIR-II photon upconversion with efficiency up to 3.9% is achieved, enabling unprecedented beyond 1000 nm low-energy sunlight-driven large-volume photocatalysis.

## INTRODUCTION

Solar energy—a safe, abundant and renewable resource—has emerged as a promising substitute for conventional sources, powering applications from photovoltaics to photocatalysis [1,2]. Concurrently, it provides an ideal platform for advancing chemical transformation technologies, particularly against the backdrop of intensifying global energy crises and mounting environmental challenges [3–5]. By eliminating the need for non-renewable energy inputs, sunlight-driven photocatalysis significantly enhances process sustainability while reducing energy costs. Crucially, this approach enables substantial reductions in carbon emissions, addressing green chemistry principles and contributing to carbon neutrality goals. Significant progress has been made in visible light-driven photocatalysis, and recent studies have extended the spectral response range to 800 nm [6,7]. However, unlocking its full potential necessitates innovative paradigms to capture and utilize untapped spectral regions, especially the near-infrared-II (NIR-II, beyond 1000 nm) region, which covers over 20% of solar energy [8]. Furthermore, NIR-II light exhibits deep solution penetration because it avoids substrate absorption [9]. As a result, NIR-II light-driven photochemical reactions are an efficient approach for expanding reaction volumes to facilitate the industrial transformation. Unfortunately, conventional materials have weak absorption in the NIR-II region, and in addition, the low energy of NIR-II photons limits the formation of high-energy excited states in photocatalysis, making direct use of these photons a challenge [10,11].

Photon upconversion, which converts low-energy photons to high-energy photons, provides a simple and straightforward strategy to utilize low-energy photons [12–14]. In particular, triplet–triplet annihilation upconversion (TTA-UC) with a high upconversion efficiency, low excitation intensity and tunable wavelengths has already demonstrated unique and promising applications such as solar cells [15,16], photocatalysis [7,17] and stereoscopic 3D printing [18,19]. Therefore, the development of a high-performance NIR-II TTA-UC system will enable unprecedented over-1000 nm sunlight-driven photocatalysis. To date, metal complexes [20,21], metal clusters [22,23] and organic small molecules [24,25] as photosensitizers, paired with suitable annihilators, have demonstrated superior upconversion performance across the visible to near-infrared spectral range. Furthermore, quantum dot (QD)-based TTA-UCs have achieved notable advances under 808 nm excitation, with reported efficiencies reaching up to 21% [26]. However, within the NIR-II window, overall TTA-UC efficiencies remain low; for example, PbS/triethylsilylethynyl-anthradithiophene (TES-ADT) exhibits an upconversion efficiency below 0.1% [27]. Although our recently developed thiophene-substituted diketopyrrolopyrrole (Th-DPP) ligand with dual coordination enables efficient triplet exciton transfer via enhanced electron coupling between the QD and ligand under a small energy gap, the overall upconversion performance remains suboptimal (0.37%) [28]. The mechanism of QD-based TTA-UC proceeds through three well-defined steps. First, photoexcited QDs transfer energy to surface ligands via Dexter-type energy transfer or charge separation-mediated triplet sensitization [29,30]. Subsequently, ligands at triplet states sensitize annihilators. Finally, two triplet annihilators undergo triplet–triplet annihilation, with one annihilator returning to the ground state and the other migrating to the singlet state to emit a high-energy photon [31]. Improving the upconversion efficiency of NIR-II TTA-UC is inseparable from not only high exciton transfer efficiency from QDs to surface ligands (Φ_TET1_), but also the long triplet lifetime of surface ligands (*τ*_T_) [28,29,32]. In our previous work, although enhanced electron coupling via ligand conformation modulation enabled efficient triplet exciton transfer, the associated intensification of the heavy-atom effect significantly shortened the triplet lifetime of the ligands. This limitation restricted their ability to sensitize the annihilator, ultimately capping upconversion performance [28]. As a result, there is an urgent need to develop new strategies to balance the triplet exciton transfer efficiency with the triplet lifetime of the ligand for efficient NIR-II TTA-UC.

**Figure 1.**
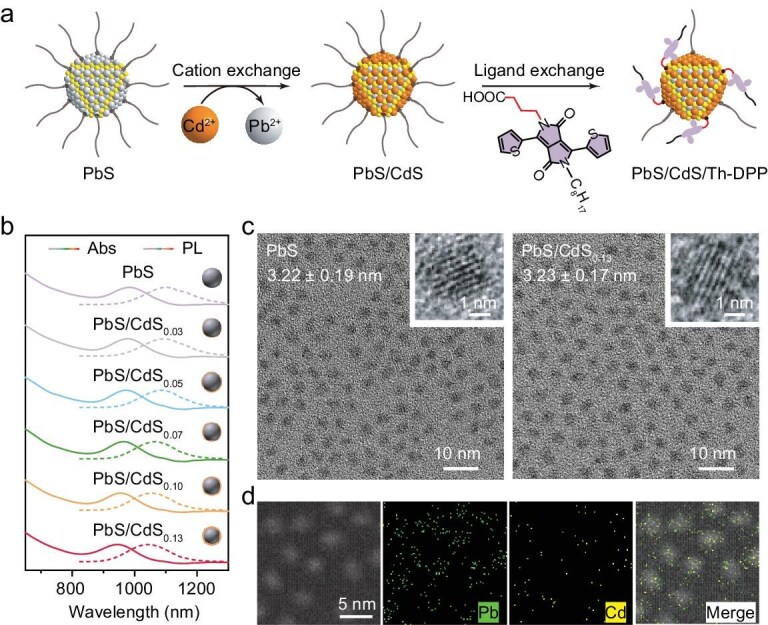
Preparation and characterization of NIR-II photosensitizers. (a) Synthesis scheme of QDs/Th-DPP NIR-II photosensitizers. (b) The absorption (solid line) and emission spectra (dashed line) of PbS QDs and PbS/CdS QDs synthesized via cation exchange in 1 μM toluene. (c) HRTEM images of PbS QDs and PbS/CdS_0.13_ QDs. The insets show the magnified view of the corresponding QD. (d) EDS elemental mapping of PbS/CdS_0.13_.

Through a precisely controlled cation-exchange approach, we constructed a CdS shell with tunable thicknesses on the PbS QDs, followed by ligand exchange to anchor the ligands (Th-DPP), creating hybrid QDs/Th-DPP NIR-II photosensitizers (Fig. 1a). Systematic characterization demonstrates that as the CdS shell thickness increases, the Th-DPP’s triplet lifetime is substantially prolonged from 0.47 to 7.8 μs, while the triplet exciton transfer efficiency decreases from 73.7% to 16.8%. By precisely optimizing the CdS shell thickness, we achieved an optimal balance between ligand triplet lifetime (2.3 μs) and triplet exciton transfer efficiency (51.4%), thereby enhancing the overall sensitization performance of the hybrid photosensitizer. Using rubrene as the annihilator, the upconversion materials demonstrated exceptional NIR-II-to-visible upconversion with a record efficiency of 3.9%. On this basis, we developed an unparalleled technology for large-volume photocatalysis driven only by sunlight beyond 1000 nm for both free radical polymerization and atom transfer radical polymerization. This work not only establishes new benchmarks for NIR-II photon upconversion but also provides a versatile platform for solar energy harvesting and conversion.

## RESULTS AND DISCUSSION

### Preparation of NIR-II photosensitizers

Through optimized synthesis of PbS QDs, we achieved high-performance QDs with a photoluminescence quantum yield of 70.7% in degassed toluene (Table S1). Steady-state spectroscopic measurements showed the first exciton absorption peak at 983 nm and the emission maximum at 1103 nm (Fig. 1b, Table S1). Using the Tauc Plot method, the bandgap was determined to be 1.16 eV. Time-resolved photoluminescence (TRPL) spectroscopy further demonstrated a monoexponential decay with a lifetime of 2.1 μs (Fig. S1). High-resolution transmission electron microscopy (HRTEM) characterization revealed monodisperse QDs with an average diameter of 3.22 nm, and distinct lattice fringes were observed (Fig. 1c). We employed a cation-exchange strategy, where Cd^2+^ ions replaced surface Pb^2+^ ions of PbS QDs, leading to the *in situ* formation of a CdS shell. By precisely controlling the amount of cadmium precursor injected, we achieved sub-monolayer precision in tuning the CdS shell thickness (Table S2). Using established literature methods [33], we calculated the average shell thicknesses as 0.03, 0.05, 0.07, 0.10 and 0.13 nm, labeling the corresponding samples as PbS/CdS_0.03_, PbS/CdS_0.05_, PbS/CdS_0.07_, PbS/CdS_0.10_ and PbS/CdS_0.13_. HRTEM analysis confirmed that PbS/CdS_0.13_ core-shell QDs retained good crystallinity and consistent diameters with PbS QDs (Fig. 1c). Energy-dispersive X-ray spectroscopy (EDS) further verified the presence of Cd (Fig. 1d). However, the CdS shell is too thin to be directly resolved, consistent with previous literature reports [34,35].

Systematic investigation of the CdS shell’s influence on PbS QDs photophysics revealed a progressive blueshift in the excitonic absorption peak, from 983 to 945 nm, with increasing shell thickness, consistent with the cation-exchange process (Fig. 1b, Table S1). Intriguingly, the photoluminescence quantum yield did not increase significantly with shell growth (Table S1), and all QD samples exhibited similar monoexponential photoluminescence (PL) decay kinetics (Fig. S1), which implied that the shell capping did not significantly affect the photoluminescence properties of QDs.

We employed Th-DPP as surface ligands, with carboxylate anchoring groups incorporated at the terminal alkyl chains for effective QD binding [28]. The ligand exchange reaction was performed at room temperature in degassed toluene solutions under argon protection. Quantitative analysis based on the molar extinction coefficient of Th-DPP revealed that surface ligand densities were around 44 per QD for the six distinct samples (Fig. S2) [28,36]. The comparable ligand loading across samples indicates that variations in CdS shell thickness did not substantially alter the ligand exchange efficiency. To investigate potential defect formation during the ligand exchange process of Th-DPP, we strategically employed 9-anthracenecarboxylic acid (ACA) as a model ligand for QD surface modification. The triplet energy level (*T*_1_) of ACA lies well above the bandgap of PbS QDs, effectively eliminating any possible exciton transfer from the QDs to the ACA. Remarkably, comparative studies revealed that the ACA-modified PbS QDs maintained both their photoluminescence intensity and monoexponential decay characteristics relative to pristine PbS QDs (Fig. S3). These observations provide definitive evidence that the ligand exchange process does not introduce surface defects.

### Effect of CdS shell thickness on triplet exciton transfer

**Figure 2.**
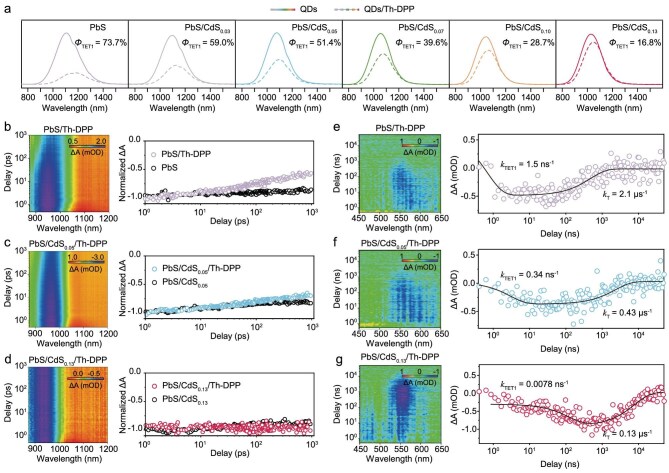
Effect of CdS shell thickness on triplet exciton transfer. (a) Relative photoluminescence intensities of QDs (solid line) and QDs/Th-DPP (dashed line). *Φ*_TET1_ represents triplet exciton transfer efficiency from QDs to Th-DPP. (b–d) 2D pseudo-colour fs-TA spectra of (b) PbS/Th-DPP, (c) PbS/CdS_0.05_/Th-DPP and (d) PbS/CdS_0.13_/Th-DPP. On the right are the corresponding QD bleach-recovery kinetics. Measurements were performed in argon-saturated toluene, λ_ex_ = 808 nm. (e–g) The double-difference ns-TA spectra within 50 μs following 1030 nm excitation of (e) PbS/Th-DPP, (f) PbS/CdS_0.05_/Th-DPP and (g) PbS/CdS_0.13_/Th-DPP after the subtraction of the QD contribution. On the right are the corresponding kinetics of Th-DPP ground-state bleaching. *k*_TET1_ represents the triplet exciton transfer rate from QDs to Th-DPP. *k*_T_ represents the triplet decay rate of Th-DPP.

Steady-state photoluminescence studies demonstrated pronounced photoluminescence quenching by Th-DPP ligands, representing the triplet exciton transfer efficiency from QDs to Th-DPP (Φ_TET1_). For PbS/Th-DPP, we observed Φ_TET1_ to be 73.7%. Systematic investigation of core-shell structures showed that increasing the CdS shell thickness to 0.13 nm progressively suppressed this quenching effect (Fig. 2a). Intriguingly, Th-DPP modification induced asymmetric emission shapes in QDs/Th-DPP hybrids compared to pristine QDs. This spectral distortion stems from the size-dependent energy landscape; smaller QDs within the ensemble exhibit stronger quenching due to their larger bandgap offset from the Th-DPP triplet energy level. This size-selective quenching highlights the delicate interplay between quantum confinement effects and interfacial energy transfer processes in these hybrid nanostructures.

Further, we utilized TRPL spectroscopy to investigate the triplet exciton transfer process (Fig. S4). After ligand exchange with Th-DPP, the photoluminescence lifetime of QDs changed from monoexponential (*τ*_0_) to biexponential decay features. In this regard, the short-lived component (*τ*_i_) is attributed to the intrinsic photoluminescence lifetime of the QDs, which is shortened due to the forward exciton transfer process from QDs to Th-DPP. The long-lived component (*τ*_d_) originated from delayed luminescence due to the reverse triplet exciton transfer. In comparison with the photoluminescence lifetimes of PbS QDs (2.1 μs), the *τ*_i_ of PbS/Th-DPP was reduced to 0.75 μs (Fig. S4). With increasing CdS thickness, the *τ*_i_ of PbS/CdS/Th-DPP was prolonged; for example, the *τ*_i_ of PbS/CdS_0.13_/Th-DPP was 1.6 μs (Fig. S4). These results agreed with the steady-state spectroscopy, suggesting that the CdS shell suppressed the exciton transfer between QDs and Th-DPP.

To elucidate the exciton transfer dynamics from QDs to surface-anchored Th-DPP ligands, we employed femtosecond transient absorption (fs-TA) spectroscopy (Fig. 2b–d and Fig. S5). Comparative studies between pristine PbS QDs and Th-DPP-functionalized counterparts revealed a pronounced acceleration of QD exciton bleach recovery upon ligand attachment, providing direct evidence of exciton transfer from the QDs to the organic ligands (Fig. 2b). Remarkably, this interfacial energy transfer process exhibited strong shell-thickness dependence. In PbS/CdS_0.05_/Th-DPP, the exciton bleach recovery showed significant retardation compared to PbS/Th-DPP (Fig. 2c). Most strikingly, when the CdS shell thickness reached 0.13 nm, the exciton recovery kinetics of PbS/CdS_0.13_/Th-DPP became virtually indistinguishable from that of PbS/CdS_0.13_ without Th-DPP (Fig. 2d). These observations unambiguously demonstrate that thicker CdS shells effectively inhibit exciton transfer across the hybrid interface.

In addition to efficient triplet exciton transfer from QDs to ligands, the extended triplet lifetime of surface ligands plays a pivotal role in facilitating triplet energy transfer to annihilators [37]. Previous studies demonstrated that while enhanced QD–ligand electronic coupling promotes exciton transfer, the heavy-atom effects drastically reduce ligand triplet lifetimes [28,32]. This is particularly critical for NIR-II TTA-UC, where intrinsically short *T*_1_ lifetimes of surface ligands—dictated by energy-gap laws—are further compromised by the complex QDs surface environment, leading to suboptimal UC performance [38–40].

We employed nanosecond transient absorption (ns-TA) spectroscopy to quantitatively determine both exciton transfer rates (*k*_TET1_) and triplet decay rates (*k*_T_). In the ns–μs time scale, a pulsed laser at 1030 nm excited the QDs, and we observed a ground-state bleaching (GSB) signal of Th-DPP at around 500–600 nm (Fig. S6), representing the triplet Th-DPP [28,41]. Since the excited-state absorption (ESA) signal of QDs mixes with the GSB signal of Th-DPP in the visible region, we subtracted the contribution of QDs from this mixed signal to extract the characteristic signal of Th-DPP and visually analyze the obtained double-difference spectra (Fig. 2e–g). We employed the Th-DPP GSB process at 565 nm to fit the *k*_TET1_ and *k*_T_ in various situations.

For PbS/Th-DPP, kinetic analysis revealed an exciton transfer rate of 1.5 ns^−1^ and Th-DPP triplet decay of 2.1 μs^−1^ (Fig. 2e). Strikingly, increasing the CdS shell thickness to 0.05 nm reduced the QD-to-Th-DPP exciton transfer rate to 0.34 ns^−1^ while slowing down the triplet decay rate of Th-DPP to 0.43 μs^−1^ (Fig. 2f). The PbS/CdS_0.13_/Th-DPP exhibited even more pronounced effects; the exciton transfer rate decreased further to 0.0078 ns^−1^, whereas the triplet decay rate of Th-DPP was significantly decelerated to 0.13 μs^−1^ (Fig. 2g). These findings establish that precise CdS shell thickness control provides a powerful strategy for regulating exciton transfer dynamics and critically extending triplet lifetime of surface ligand, which is a key requirement for advanced TTA-UC applications.

As the CdS shell thickness increases, the triplet exciton transfer efficiency decreases due to the confinement of the PbS core wavefunction [42]; meanwhile, the triplet lifetime of Th-DPP is prolonged as a result of the weaker heavy-atom effect of Cd compared to Pb. We have also demonstrated the influence of the CdS shell on ligand conformation. The interaction between metal ions on the QD surface and sulfur atoms in the Th-DPP ligand plays a critical role in governing ligand conformation, promoting ligand proximity to the QD surface. Using 800 MHz nuclear magnetic resonance (NMR), we analyzed the conformation of Th-DPP on the QD surface. When the thiophene group of the ligand approaches the QD surface, the chemical shifts of its protons are affected by surface metal ions. In the PbS/Th-DPP, the proton chemical shift on one side of DPP shifted from 8.93 to 8.85 ppm, while the opposite side remained nearly unchanged (Fig. S7), indicating that Th-DPP preferentially adopts a conformation with one thiophene group oriented toward the PbS QD surface, consistent with previous reports [28]. In contrast, in the PbS/CdS_0.13_/Th-DPP, this chemical-shift perturbation was markedly reduced (Fig. S7). According to the hard–soft acid–base principle, Cd^2+^ interacts more weakly with S^2−^ than Pb^2+^, resulting in insufficient interaction to stabilize Th-DPP in proximity to the QD surface.

To further support our findings, molecular dynamics simulations were performed. Based on the actual size of the QD and the number of surface ligands, a model was constructed featuring a QD approximately 3.2 nm in diameter, capped with 35 oleic acid molecules and 35 Th-DPP ligands. For the PbS/CdS/Th-DPP, Pb^2+^ ions on the PbS QD surface were replaced by Cd^2+^. After optimizing the conformation of Th-DPP on the QD surface, the radial distribution function of the thiophene groups relative to the QD center was analyzed. In PbS/Th-DPP, the Th-DPP ligands exhibit a relatively narrow distribution with an average distance of approximately 17.6 Å from the PbS QD center (Fig. S8). In contrast, for PbS/CdS/Th-DPP, the primary distribution peak shifts to 18.5 Å, the distribution broadens significantly, and a substantial fraction of ligands are located at 21 Å or further from the surface (Fig. S8). These results indicate that, owing to the weaker Cd-S interaction, Th-DPP cannot maintain a stable conformation near the QD surface, resulting in an increased average distance from the surface. This effect weakens the coupling between Th-DPP and the PbS QD, which suppresses triplet energy transfer efficiency while prolonging the triplet lifetime of the ligand. Thus, these synergistic effects balance triplet exciton transfer efficiency with ligand triplet lifetime, thereby enhancing the overall sensitization performance of the hybrid photosensitizer. Notably, the weaker surface binding may shift ligand conformation from a static stable state to a dynamically switching mode. This mechanism provides a more favorable dynamic scenario, where the ligand achieves efficient triplet exciton transfer via strong close-range coupling in the ground state, then moves away from the QD surface to suppress the heavy-atom effect, thereby maintaining a prolonged triplet lifetime.

### NIR-II excitable photon upconversion

**Figure 3.**
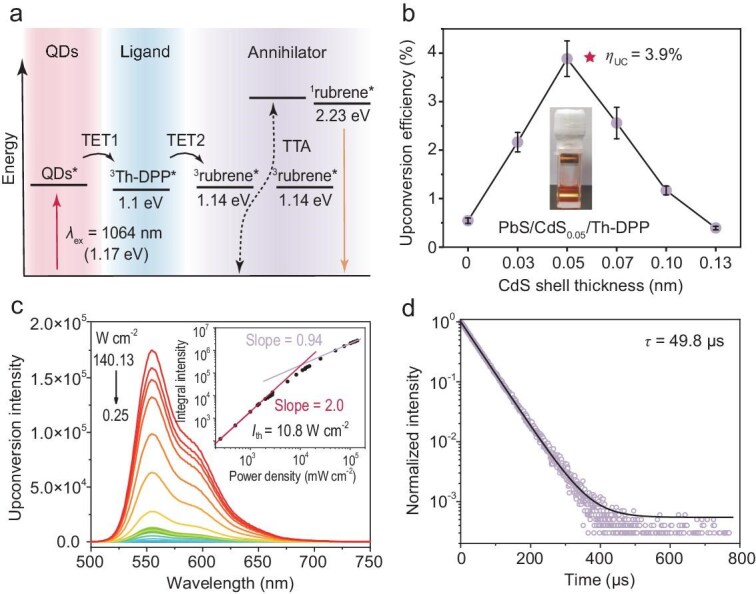
Efficient near-infrared-II excitable photon upconversion based on QDs. (a) Schematic of the NIR-II TTA-UC system containing QDs/Th-DPP and rubrene. QDs*, ^3^Th-DPP*, ^3^rubrene* and ^1^rubrene* denote the excited state of QDs, the *T*_1_ state of Th-DPP, the *T*_1_ state of rubrene and the *S*_1_ state of rubrene; TET1, TET2 and TTA denote the triplet exciton transfer from QDs to Th-DPP, Th-DPP to rubrene, and triplet–triplet annihilation of rubrene. (b) Effect of CdS shell thickness on upconversion efficiency. The red star marks the photon upconversion with the highest efficiency reported so far, using PbS/CdS_0.05_/Th-DPP as the photosensitizer, and the inset is its photograph under 1064 nm laser irradiation in an airtight cuvette. (c) Power-dependent upconversion spectra of PbS/CdS_0.05_/Th-DPP/rubrene with various power densities (0.25–140.13 W cm^–2^) at 1064 nm. The inset is the dependence of the upconversion intensity on the incident power density at 1064 nm, *I*_th_ = 10.8 W cm^–2^. (d) Time-resolved upconversion luminescence decay curve of PbS/CdS_0.05_/Th-DPP/rubrene under 1064 nm excitation. Measurements were performed at 10 μM QDs/Th-DPP and 20 mM rubrene.

Leveraging the *T*_1_ of Th-DPP, we selected energetically matched rubrene as annihilator to systematically evaluate NIR-II TTA-UC performance across CdS shell thicknesses (Fig. 3a). The upconversion efficiency (*η*_UC_) is related to the intersystem crossing quantum yield (Φ_ISC_) of the QDs, the triplet exciton transfer efficiency from QDs to Th-DPP (Φ_TET1_) and from Th-DPP to rubrene (Φ_TET2_), the triplet–triplet annihilation of rubrene (Φ_TTA_) and the fluorescence quantum yield of rubrene (Φ*_f_*). The strong spin–orbit coupling of QDs causes the Φ_ISC_ to approach 1. Both Φ_TTA_ and Φ*_f_* depend on rubrene itself. Thus, the two-step triplet exciton transfer process determines the upconversion performance [43].

We tested the photon upconversion under 1064 nm illumination. As shown in Fig. 3b, *η*_UC_ exhibited a non-monotonic dependence on shell thickness, peaking at 0.05 nm with a value of 3.9%, representing a 10-fold enhancement compared to the highest value reported in the literature (Table S3) [28]. Intriguingly, further increasing CdS thickness led to pronounced *η*_UC_ reduction. This critical transition reveals that while thicker shells prolong Th-DPP’s *T*_1_ lifetimes (as demonstrated earlier), the QD-to-Th-DPP exciton transfer rate ultimately becomes the dominant limiting factor for UC performance. As previously analyzed, the precisely engineered CdS shell balances triplet exciton transfer efficiency with ligand triplet lifetime, thereby enhancing the overall sensitization performance of the hybrid photosensitizer and consequently improving upconversion efficiency. We then evaluated the stability of the upconversion luminescence. Under continuous 1064 nm laser (15 W cm^–2^) illumination for 60 min, no attenuation in upconversion intensity was observed (Fig. S9). This excellent photostability arises from the intrinsic photobleaching resistance of the PbS QDs, thereby providing a viable pathway toward the development of durable long-wavelength photocatalysis.

Further, we tested the power-dependent upconversion luminescence of PbS/CdS_0.05_/Th-DPP/rubrene (Fig. 3c). The upconversion intensity showed a quadratic-to-linear transition with respect to the incident power density. We observed a lower threshold power density (*I*_th_) of 10.8 W cm^–2^ under 1064 nm illumination, compared to the reported results, e.g. 43 W cm^–2^ for PbS/TES-ADT [27] and 23.5 W cm^–2^ for PbS/Th-DPP [28]. The low *I*_th_ contributes to the advancement of photon upconversion materials for photovoltaic devices and photocatalytic applications. Moreover, the microsecond upconversion lifetimes further confirmed that the yellow emission results from the TTA mechanism (Fig. 3d). In addition, the PbS/CdS_0.05_/Th-DPP/rubrene exhibits an anti-Stokes shift of 1.07 eV, which approaches the theoretical limit of the anti-Stokes shift (1.17 eV). We have summarized recent progress in NIR-II TTA-UC and compared key performance parameters in Table S3. The low upconversion efficiency continues to be a critical bottleneck limiting practical applications of TTA-UC materials. In this work, we constructed an NIR TTA-UC system that achieved a record upconversion efficiency of 3.9% under 1064 nm excitation, coupled with a large anti-Stokes shift and low threshold power density, indicating a significant advancement.

### Beyond-1000 nm low-energy sunlight-driven photocatalysis

**Figure 4.**
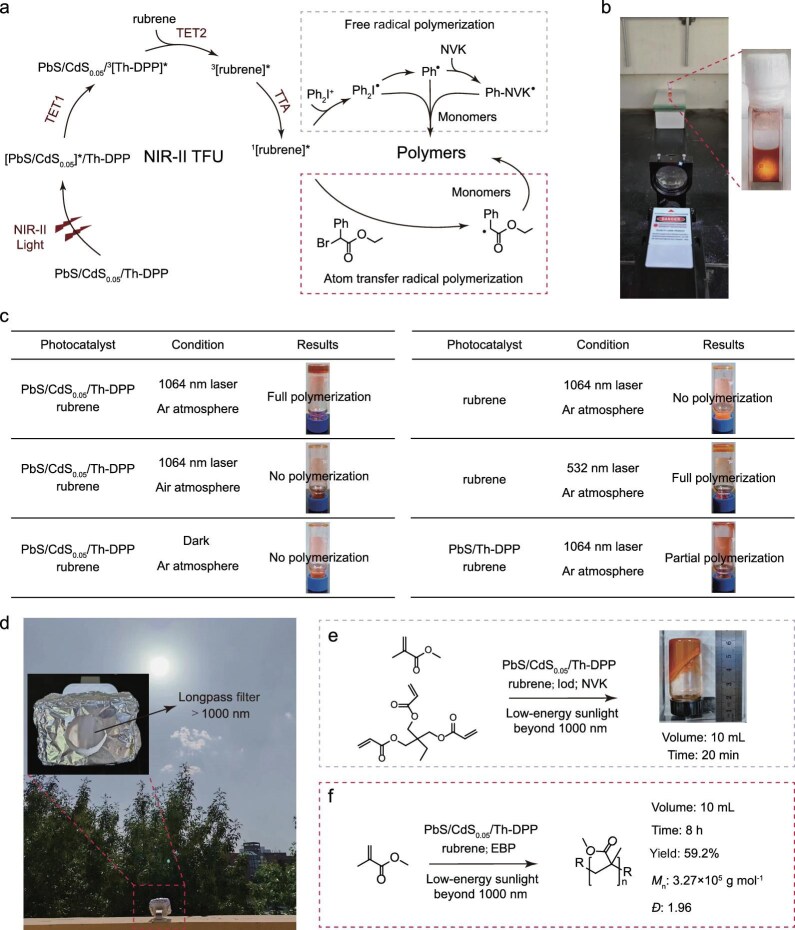
Photocatalysis driven by low-energy NIR-II photons. (a) A proposed mechanism for free radical polymerization and ATRP mediated by PbS/CdS_0.05_/Th-DPP/rubrene. (b) Diagram of the device for 1064 nm laser-driven photopolymerization. The upconversion system (PbS/CdS_0.05_/Th-DPP/rubrene) in an airtight cuvette was used to determine the spot size (9 mm, 1 W cm^–2^). The power density of the laser was then adjusted to 0.1 W cm^–2^, and a glass vial was used in place of the cuvette for the organic transformations. (c) Summary table of the experimental conditions and outcomes of free radical polymerization driven by a 1064-nm laser (0.1 W cm^–2^). (d) Diagram of a device for beyond-1000 nm low-energy sunlight-driven photopolymerization. The reaction vessel was placed in a square box wrapped in aluminum foil with a longpass filter (>1000 nm) in the middle to acquire low-energy sunlight beyond 1000 nm. (e and f) The experimental condition and result of (e) free radical polymerization and (f) ATRP.

Capitalizing on our high-performance NIR-II TTA-UC materials, we developed a groundbreaking photocatalysis platform driven by low-energy NIR-II photons. The study strategically employed both free radical polymerization and atom transfer radical polymerization (ATRP) as model reactions to demonstrate the unique capabilities of NIR-II photochemistry (Fig. 4a). We conducted the free radical polymerization of acrylates involving the monomers trimethylolpropane triacrylate (TMPTA) and methyl methacrylate (MMA), with diphenyliodonium (Iod) and *N*-vinylcarbazole (NVK) as the initiators to initiate the reaction [7]. The reaction setup includes a light source of a 1064 nm laser and a lens in the center to adjust the spot (Fig. 4b). Using PbS/CdS_0.05_/Th-DPP/rubrene as photocatalyst, significant free radical polymerization was achieved with low excitation intensity (100 mW cm^–2^ at 1064 nm for 20 min duration) (Fig. 4c). Control experiments confirmed the essential role of the *S*_1_ state of rubrene (^1^rubrene*), where no polymerization occurred in an air atmosphere, under dark conditions, or with rubrene/1064 nm illumination, while rubrene/532 nm illumination successfully initiated polymerization (Fig. 4c). It is worth noting that only a small proportion of monomers were polymerized under the same experimental conditions using PbS/Th-DPP/rubrene with suboptimized upconversion performance as photocatalyst, which confirms the importance of developing high-performance upconversion materials (Fig. 4c).

The broadband harvesting of NIR-II photons by PbS/CdS_0.05_/Th-DPP has motivated us to utilize beyond-1000 nm low-energy sunlight to drive photocatalysis. We constructed the reaction setup as shown in Fig. 4d. The reaction vessel was placed in a square box wrapped in aluminum foil with a longpass filter (>1000 nm) in the middle to acquire low-energy sunlight beyond 1000 nm (Fig. S10). When exposed to sunlight for 5 min, PbS/CdS_0.05_/Th-DPP/rubrene completely polymerizes the monomers in a reaction volume of 200 μL (Fig. S11). The addition of only rubrene as a photocatalyst resulted in no photopolymerization, proving the reliability of this complex in conducting low-energy sunlight-driven polymerization (Fig. S11). Thanks to the deep solution penetration of NIR-II light, we achieved remarkable bulk-free radical polymerization in 10 mL volumes, with significant polymerization observed after just 20 min of sunlight irradiation (Fig. 4e). In addition, we tried beyond-1000 nm low-energy sunlight-driven ATRP for precision polymerization in a large volume (10 mL) using MMA as the monomer and ethyl α-bromophenylacetate (EBP) as the initiator. After 8 h of sunlight exposure, 59.2% monomer conversion was attained (Fig. 4f, Figs S12 and S13). The high polymer dispersity index (*Đ* = 1.96) likely arises from two factors: (i) oxygen permeation during the prolonged reaction, causing partial catalyst deactivation under static oxygen conditions; and (ii) increased viscosity as the reaction progresses, which restricts the mobility of polymerization sites and promotes localized overpolymerization, ultimately broadening the *Đ* of the resulting polymer. Additionally, following the reaction, the QDs become embedded within the polymer matrix, rendering catalyst recovery unfeasible under the current conditions. In future studies on other photocatalytic systems, we will pursue and optimize strategies for catalyst recovery to enhance sustainability and application potential [44,45]. Regardless, we achieved unprecedented beyond-1000 nm low-energy sunlight-driven large-volume photocatalysis through optimizing QD shell thickness to enhance NIR-II TTA-UC.

## CONCLUSION

QD-based photon upconversion presents a compelling approach for direct utilization of the NIR-II spectral window, with the central challenge lying in precise QD surface engineering to enhance exciton transfer performance, encompassing efficient exciton transfer and extended triplet lifetime of ligands. In this work, we developed an innovative shell-thickness modulation strategy to enhance the overall sensitization performance of hybrid photosensitizers. This approach, however, presents a fundamental trade-off; while thicker shells prolong triplet lifetimes, they concurrently diminish exciton transfer efficiency, creating an intrinsic optimization barrier. Through systematic investigation, we identified an optimal CdS shell thickness of 0.05 nm to achieve this critical balance, yielding a record upconversion efficiency of 3.9%, which is a 10-fold improvement over previous benchmarks. The developed NIR-II TTA-UC enabled unprecedented beyond-1000 nm low-energy sunlight-driven photocatalysis, overcoming the spectral limitations of visible-light technologies. This work not only establishes a new paradigm for precise exciton management in QD-based materials but also provides transformative solutions for solar energy harvesting and conversion.

## Supplementary Material

nwag078_Supplemental_File
