# Supplementary material for: Beyond-1000 nm low-energy sunlight-driven photocatalysis enabled by quantum dot-based photon upconversion
Source: Natl Sci Rev. 2026 Feb 5;13(7):nwag078. doi: 10.1093/nsr/nwag078 (PMC13123524; doi:10.1093/nsr/nwag078)
Supplement: nwag078_Supplemental_File [file nwag078_supplemental_file.pdf]

Supporting Information

Beyond 1000 nm low-energy sunlight-driven photocatalysis enabled by quantum dot-based photon upconversion

Lin-Han Jiang[1], Ming-Yu Zhang[1], Jia-Yao Li[1], Ran Li[1], Ying-Ze Li[1], Hong-Juan Feng[1], Weijian Sun[2], Xiaofei Miao[3], Junxue Liu[2], Wenbo Hu[3], Ling Huang[1,*], and Dai-Wen Pang[1,*]

[1]Frontiers Science Center for New Organic Matter, Research Center for Analytical Sciences, College of Chemistry, State Key Laboratory of Medicinal Chemical Biology, Tianjin Key Laboratory of Biosensing and Molecular Recognition, Frontiers Science Center for Cell Responses, Haihe Laboratory of Sustainable Chemical Transformations, Nankai University, Tianjin 300071, China;

[2]Time-Tech Spectra Co., Ltd., Dalian 116085, China;

[3]Frontiers Science Center for Flexible Electronics and Xi'an Institute of Flexible Electronics (IFE), Northwestern Polytechnical University, Xi'an 710072, China.

*Corresponding authors. E-mails: dwpang@whu.edu.cn; huangl1@nankai.edu.cn

This PDF file includes:

Supporting Text
Tables S1 to S3
Figs S1 to S13
Supporting References

1. Supporting Text

1.1 Chemicals

All the mentioned chemicals were used as received without further purification unless otherwise noted. Sodium, tetrahydrofuran (THF), oleic acid (OA), oleylamine, 1-octadecene (ODE), bis(trimethylsilyl)sulfide (TMS_2S), cadmium acetate dihydrate, trimethylolpropane triacrylate (TMPTA), and ethyl α-bromophenylacetate (EBP) were purchased from Shanghai Aladdin Biochemical Technology Co., Ltd. 2-Methyl-2-butanol was purchased from Adamas-Beta. Anhydrous ferric chloride was purchased from RHAWN. 2-cyanothiophene, potassium iodide, ethyl bromoacetate, ethyl 4-bromobutyrate, ethyl 8-bromooctanoate, diphenyliodonium (Iod), and N-vinylcarbazole (NVK) were purchased from Shanghai Bide Pharma Co. Ltd. 18-crown-6 ether was purchased from Shanghai Jiuding Chemical Technology Co. Potassium carbonate (K_2CO_3) was purchased from BENCHMARK. Bromooctane, lithium hydroxide monohydrate ($LiOH \cdot H_2O$), and rubrene were purchased from MERYER. N, N-dimethylformamide (DMF) was purchased from J&K Scientific. Diisopropyl succinate, acetic acid, and methyl methacrylate (MMA) were purchased from TCI. Deuterated chloroform ($CDCl_3$) and dimethyl sulfoxide-d6 ($(CD_3)_2SO$) were purchased from Innochem. Lead oxide (PbO) was purchased from Alfa Aesar. Toluene was purchased from Tianjin Bohua Chemical Reagent. Methanol (MeOH), ethyl acetate (EtOAc), ethanol (EtOH), dichloromethane (DCM), hexane, and silica gel (40–63 μm) were purchased from the Tianjin Hedong District Guangda Service Department.

1.2 Characterization

^{1}H NMR spectra were recorded on a Bruker AVANCE III 400 MHz spectrometer and an AVANCE NEO 800 MHz spectrometer. UV-vis absorption spectra were recorded by a Shimadzu UV-3600 plus spectrophotometer. A Talos F200X G2 high-resolution transmission electron microscope (HRTEM) was used for the acquisition of HRTEM images. Energy-dispersive X-ray spectroscopy (EDS) were performed on Aberration Corrected Transmission Electron Microscope (JEM-ARM200F). Photoluminescence (PL) spectra, PL quantum yields, and transient PL spectra were measured on an Edinburgh FLS1000 spectrometer. The transient absorption (TA) spectra were collected by an ultrafast transient absorption spectrometer (Tsunami Oscillator, 80 MHz, 808 nm) and nanosecond transient absorption spectroscopy (Nano-TA100). Gel permeation chromatography was recorded on a Hitachi L-2490.

1.3 Synthetic procedure of PbS/CdS QDs

PbS QDs were synthesized based on literature [1]. A mixture of PbO (0.45 g), oleic acid (2 mL), and ODE (18 mL) was heated to 110 °C in a 50 mL three-neck flask for an hour. Meanwhile, the sulfur precursor was prepared by mixing bis(trimethylsilyl)sulfide (0.21 mL) in anhydrous ODE (10 mL). The sulfur precursor was swiftly injected and the heater removed right after injection. 2 min after injection, the reaction flask was rapidly cooled down to room temperature and the injection of cold hexane (10

mL). As synthesized PbS QDs were washed three times by adding 1:3 volume hexanes/ethanol mixture, followed by centrifuging at 8000 rpm for 5 min. The supernatant was discarded and the final pellet was quickly transferred to a glovebox and redispersed in toluene for future use.

PbS/CdS QDs were synthesized as follows. The cadmium precursor was made by addition of 66.7 mg cadmium acetate dihydrate into 0.814 mL ODE and 0.187 mL oleylamine. Then, the precursor was heated at 120 °C for 30 min until a clear solution formed. The different aliquots of cadmium precursor were added to 1 mL PbS QDs (100 μM in toluene) while stirring at 85 °C. After 7 min, 100 μL oleic acid was injected followed by the injection of 900 μL hexane to quench the reaction. Then particles were cleaned once with addition of 10 mL acetone. Finally, the QDs were transferred to a glovebox and redispersed in toluene. The size of the PbS core was determined by the position of the first exciton peak, from which the molar extinction coefficient at 400 nm was calculated to obtain the concentration of QDs [2].

1.4 The measurement of PLQY for QDs.

Our equipment as well as the absolute method for testing the PLQY were calibrated by engineers from Edinburgh Instruments Ltd. The measurement of PLQY for QDs is an absolute method, with three independent experiments performed. 808 nm laser (MDL-III-808-2W from Changchun New Industries Optoelectronics Technology Co., Ltd.) was used as the light source, and an lnGaAs detector (G12182-230K from Hamamatsu) was used to collect the signal, and an integrating sphere (SM4 from Edinburgh Instruments Ltd.) was used for testing. Specifically, toluene was added to a 1 mm × 1 cm cuvette to collect the spectral signal of the blank solution. Under the same conditions, the spectral signal of the sample solution (1 μM QDs in argon-saturated toluene) was tested. Finally, the absolute PLQY of QDs was obtained by the Edinburgh Instrument's own software.

1.5 Calculation of CdS shell thickness from ICP-OES analysis

The CdS shell was formed through a cation-exchange process. Both the PbS/CdS QDs and the PbS core were modeled as spheres with radii R and r, respectively.

$$V_{PbS} = \frac{4}{3}\pi r^3, \quad V_{CdS} = \frac{4}{3}\pi(R^3 - r^3)$$

$$a = \frac{n_{Pb}}{n_{Cd}} = \frac{n_{PbS}}{n_{CdS}} = \frac{\frac{V_{PbS} \times \rho_{PbS}}{MW_{PbS}}}{\frac{V_{CdS} \times \rho_{CdS}}{MW_{CdS}}}$$

$$r^3 = \frac{R^3}{(1 + \frac{0.94}{a})}$$

Here, V_{PbS} and V_{CdS} are the volumes corresponding to the PbS core and CdS shell. The molar ratios of the cations (a) is calculated from ICP-OES analysis. MW and ρ are the molecular weight and the density respectively.

1.6 Preparation of QDs/Th-DPP material

Ligand exchange was performed by mixing QDs with Th-DPP in argon saturated toluene and stirred for 40 min [3]. The total volume was 1 mL and the final concentrations were 10 μM for QDs and 600 μM for Th-DPP. As synthesized QDs/Th-DPP were washed by adding 1:5 volume toluene/acetone mixture, followed by centrifuging at 8000 rpm for 10 min. The supernatant was discarded and the final pellet was quickly transferred to a glovebox and redispersed in toluene for future use. The concentration of the QDs/Th-DPP was determined using the absorption at 400 nm.

1.7 Triplet exciton transfer efficiency from QDs to Th-DPP (Φ_{TET1})

The triplet exciton transfer efficiency from QDs to Th-DPP (Φ_{TET1}) was calculated using equation 1 [4]. The phosphorescence intensity of QDs/Th-DPP (I_t) and QDs (I_0) was measured.

$$\Phi_{\mathrm{TET1}} = 1 - \frac{I_t}{I_0} \quad (1)$$

1.8 The transient absorption spectra (TAS) analysis

Femtosecond transient absorption spectra (fs-TAS) were processed in the Surface Xplorer software for background subtraction and chirp correction. The resulting data were plotted in Origin, from which the kinetics at the exciton bleach region of the quantum dots were extracted. Nanosecond transient absorption spectra (ns-TAS) were treated for noise subtraction using TAS Analyzer and subsequently plotted in Origin. Since the excited-state absorption (ESA) signal of QDs mixes with the GSB signal of Th-DPP in the visible region, we subtracted the contribution of QDs from this mixed signal to extract the characteristic signal of ^{3}Th-DPP* and visually analyze the obtained double-difference spectra [5]. Specifically, the ns-TA spectra of oleic acid-covered QDs at 1 ns was taken as the characteristic spectra of the QDs. The contribution of QDs in QDs/Th-DPP is derived from the ratio of the average of the characteristic spectra of QDs at 740-750 nm to the corresponding average of the spectra of QDs/Th-DPP at different delay times. The double-difference spectra reflecting ^{3}Th-DPP* is obtained by subtracting the two. The rise and decay of this signal corresponded to the formation of ^{3}Th-DPP* via triplet exciton transfer and its subsequent decay, respectively. The kinetic traces extracted at 550 nm were fitted with a biexponential decay model, with the resulting time constants corresponding to the triplet exciton transfer rate and the triplet decay rate, respectively.

1.9 Atomistic molecular dynamics simulations

Atomistic molecular dynamics simulations have been performed in the GROMACS (version 2020.6) simulation package [6]. The Universal force field, which cover the whole periodic table, was used for the quantum dot and the General Amber force field (GAFF2) was used for the oleic acid and the Th-DPP ligands. A globular quantum dot of diameter ~3.2 nm was built from the crystal structure of PbS unit cell and decorated with 35 oleic acid and 35 Th-DPP molecules on the surface. For the PbS/CdS/Th-DPP, Pb^{2+} ions on the PbS QD surface were replaced by Cd^{2+}. The quantum dot was first placed at the center

of a cubic box of around 8 nm and thousands of steps of energy minimization was performed before solvation of the preequilibrated methyl benzene molecules. After thousands of steps of energy minimization, equilibration of 5 ns was performed under the NPT ensemble. The production molecular dynamics simulations were performed for 50 ns under the canonic ensemble. The temperature was coupled to 298 K using the Nose-Hoover method and the cutoff scheme of 1.2 nm was implemented for the non-bonded interactions. The Particle Mesh Ewald method with a fourierspacing of 0.1 nm was applied for the long range electrostatic interactions [7]. All covalent bonds with hydrogen atoms were constraint using the LINCS algorithm [8]. Radial distribution function of thiophene moieties near the carboxylic acid from the QD center was determined from dynamics simulations results.

1.10 Correction of UC spectra

Due to the high concentration of rubrene and the strong absorption of QDs/Th-DPP in the visible region, we corrected the UC spectra with the fluorescence spectra of rubrene in diluted solution and obtained the corrected UC spectra excluding the inner-filtering effect [9]. Specifically, we multiplied the intensity ratio of the UC spectra to the dilute solution rubrene spectra at 650 nm, where the influence of inner-filtering effects is negligible, to the dilute solution rubrene spectra, and thus simulated the corrected UC spectra.

1.11 The corrected upconversion efficiency (η_{uc}, normalized to 100%)

The η_{uc} was calculated by comparison with the standard 2,6-diiodo-BODIPY(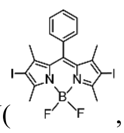

, Φ_{std} = 2.7% in acetonitrile, [10]) via equation 2 and 3 [3].

$$\eta_{uc} = 2\times\Phi_{ref}\times(\frac{A_{std}}{A_{sam}})\times(\frac{I_{sam}}{I_{std}})\times(\frac{n_{sam}}{n_{std}})^2 \text{...}(2)$$

$$A = \frac{\text{Laser Power}}{hc/\lambda}(1 - 10^{-Abs}) \text{...}(3)$$

The A_{sam}/A_{std} represent the number of photons absorbed by the sample/standard, respectively. The I_{sam} was the integrated intensity of corrected UC spectra of the sample and the I_{std} represent the integrated luminescence intensity of standard. n_{sam}/n_{std} are the solvent refractive index of the sample/standard, respectively. The upconversion samples were excited at 1064 nm, and the standard was excited at 532 nm.

1.12 NIR-II-driven photocatalysis

The polymerization systems were all configured in an argon-filled glove box. For 200 µL free radical polymerization system [11], Iod (2 mg), NVK (5 mg), TMPTA (90 µL) and MMA (90 µL) were added to a vial containing PbS/$CdS_{0.05}$/Th-DPP toluene solution (20 µL) and rubrene (2.13 mg). For 10 mL free radical polymerization system, all mentioned above expanded 50 folds. For 10 mL atom-transfer radical polymerization system [12], PbS/$CdS_{0.05}$/Th-DPP toluene solution (1 mL), rubrene (106.5 mg) and EBP

(150 μL), were added to a polymerization matrix of methyl methacrylate (9 mL). The final concentration of PbS/$CdS_{0.05}$/Th-DPP and rubrene were 10 μM and 20 mM, respectively. The vials were sealed and reacted under different conditions. For excitation with a 1064 nm laser, our reaction setup consists of a 1064 nm light source and a lens to adjust the spot. The upconversion system (PbS/$CdS_{0.05}$/Th-DPP/rubrene) in an airtight cuvette was used to determine the spot size (9 mm, 1 W/cm^2). The power density of the laser was then adjusted to 0.1 W/cm^2, and a glass vial was used in place of the cuvette for photopolymerization. The reaction setup for beyond 1000 nm low-energy sunlight to drive photopolymerization was designed as follows. The reaction vessel was placed in a square box wrapped in aluminum foil with a longpass filter (>1000 nm) in the middle to acquire low-energy sunlight beyond 1000 nm.

2. Supporting Tables

Table S1. Photophysical properties of QDs.[a]

	λ_{abs}[b]	ε[c]	λ_{em}[d]	PLQY[e]
PbS	983	7.85	1103	69.9±3.3
PbS/$CdS_{0.03}$	977	7.63	1095	71.1±3.2
PbS/$CdS_{0.05}$	972	7.49	1087	68.4±1.9
PbS/$CdS_{0.07}$	964	7.28	1065	75.1±3.1
PbS/$CdS_{0.10}$	955	7.08	1057	78.6±3.1
PbS/$CdS_{0.13}$	945	6.81	1041	79.3±4.0

[a] Measured in toluene, 1 μM. [b] The first exciton absorption peak (nm). [c] Molar absorption coefficient at 400 nm ($\times 10^5$ M^{-1} cm^{-1}). [d] Maximum emission wavelength (nm). [e] The absolute photoluminescence quantum yield (%) was determined using an integrating sphere with three independent measurements.

Table S2. ICP-OES results of PbS/CdS QDs and calculated thicknesses of CdS shell.

	Cd-acetate[a]	Pb[b]	Cd[c]	CdS shell thickness[d]
PbS/$CdS_{0.03}$	4	70.9	1.99	0.03
PbS/$CdS_{0.05}$	8	85.1	4.93	0.05
PbS/$CdS_{0.07}$	16	47.8	3.86	0.07
PbS/$CdS_{0.10}$	24	59.3	7.26	0.10
PbS/$CdS_{0.13}$	32	63.0	10.8	0.13

[a] Different amounts of Cd-acetate (250 μM) used in the synthesis of PbS/CdS QDs (μL). [b] Concentration of Pb analyzed by ICP-OES (μg/mL). [c] Concentration of Cd analyzed by ICP-OES (μg/mL). [d] The calculated thicknesses of CdS shell (nm).

Table S3. Summary of reported NIR-II TTA-UC.

Photosensitizer	Annihilator	λ_{ex}[a]	Anti-Stokes shift[b]	η_{UC}[c]	I_{th}[d]	Reference
PbS	TES-ADT	975	0.76	0.06	113	ACS Appl. Nano Mater. 2021, 4, 9680-9688
Yb-L	Rubrene	980	0.95	≪1%	--	J. Phys. Chem. Lett. 2020, 11, 2477−2481
PbSe	Rubrene	980	0.92	0.003	--	Nano Lett. 2015, 15, 5552-5557
PbS	TES-ADT	1064	0.86	0.094	43	Chem. Sci. 2019, 10, 4750-4760
PbS-3.11/Th-DPP	Rubrene	1064	1.07	0.37	23.5	J. Am. Chem. Soc. 2024, 146, 10785-10797
**PbS/CdS$_{0.05}$/Th-DPP**	**Rubrene**	**1064**	**1.07**	**3.9**	**10.8**	**This work**

[a] Excitation wavelength (nm). [b] Anti-Stokes shift (eV). [c] Upconversion efficiency (%, normalized to 100%). [d] The threshold power density (W/cm^2).

3. Supporting Figures

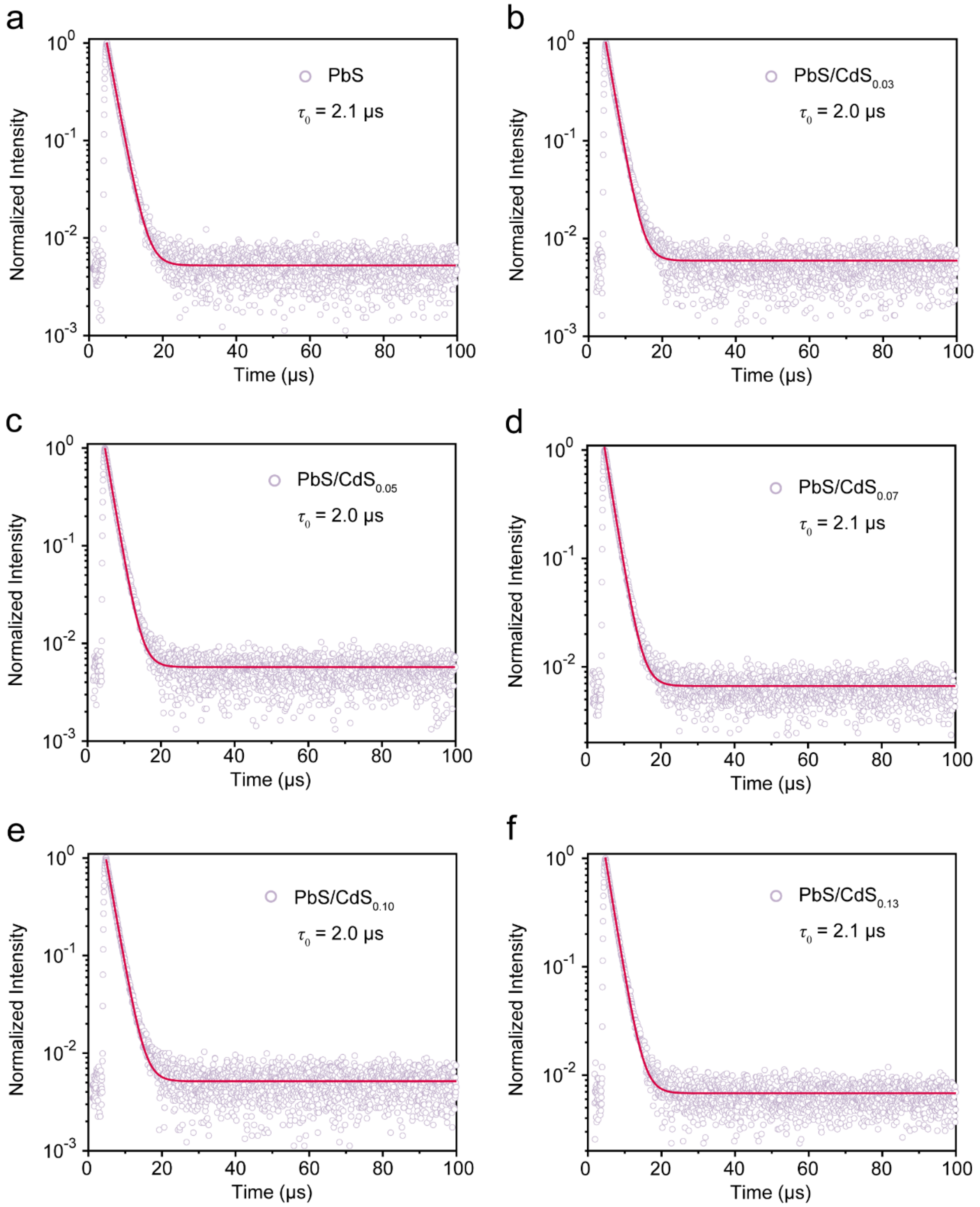

Fig. S1. Time-resolved photoluminescence spectra of QDs in argon-saturated toluene. Measurements were performed at 1 μM, λ_{ex} = 808 nm.

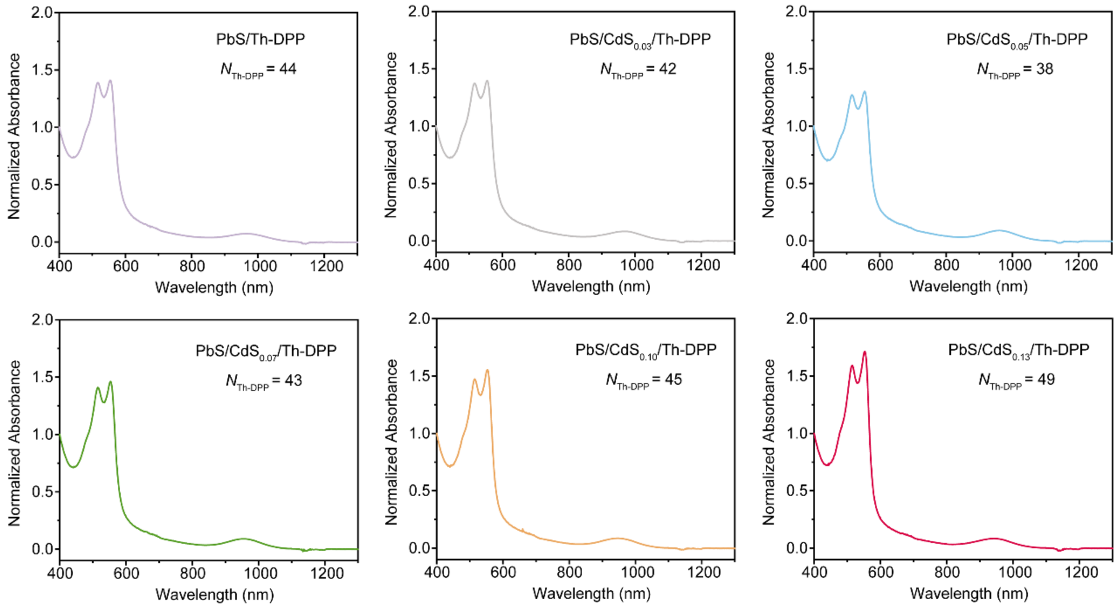

Fig. S2. The absorption spectra of QDs/Th-DPP. $\langle N_{\text{Th-DPP}} \rangle$ represents the number of Th-DPP attached to the surface of each QD.

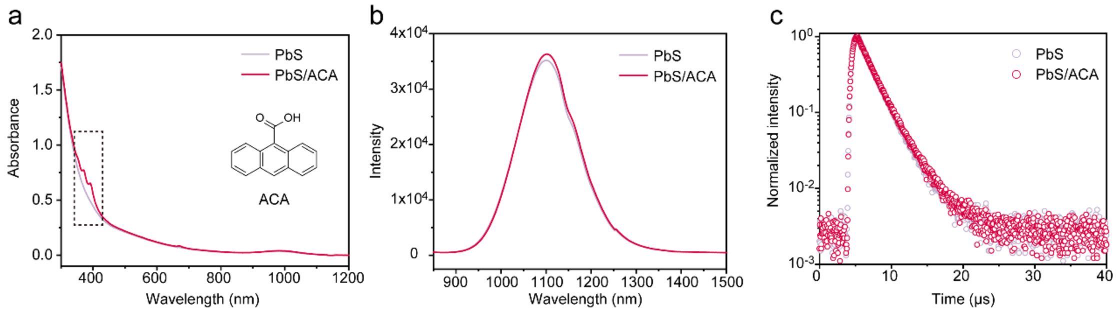

Fig. S3. (a) Absorption spectra of PbS QDs and PbS/ACA after ligand exchange with 600 μM 9-anthracenecarboxylic acid (ACA). (b) Photoluminescence spectra and (c) time-resolved photoluminescence spectra of PbS QDs and PbS/ACA in argon-saturated toluene. Measurements were performed at 1 μM, λ_{ex} = 808 nm.

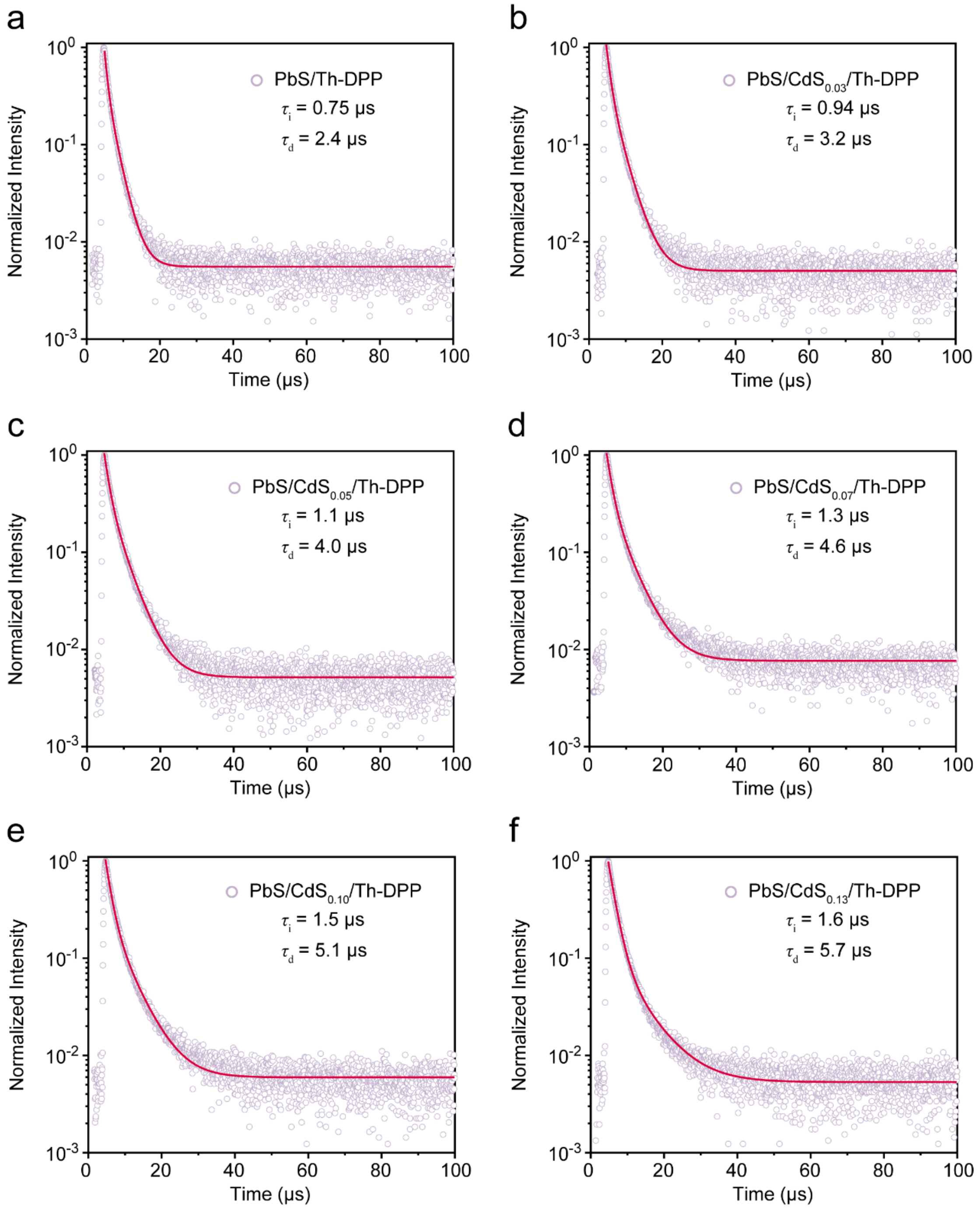

Fig. S4. Time-resolved photoluminescence spectra of QDs/Th-DPP in argon-saturated toluene. Measurements were performed at 1 μM, λ_{ex} = 808 nm.

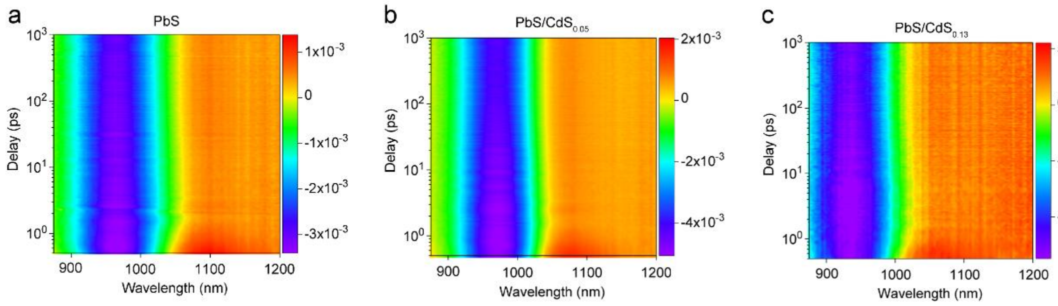

Fig. S5. Two-dimensional pseudo-colour femtosecond transient absorption spectra of (a) PbS, (b) $PbS/CdS_{0.05}$, and (c) $PbS/CdS_{0.13}$. Measurements were performed in argon-saturated toluene, λ_{ex} = 808 nm.

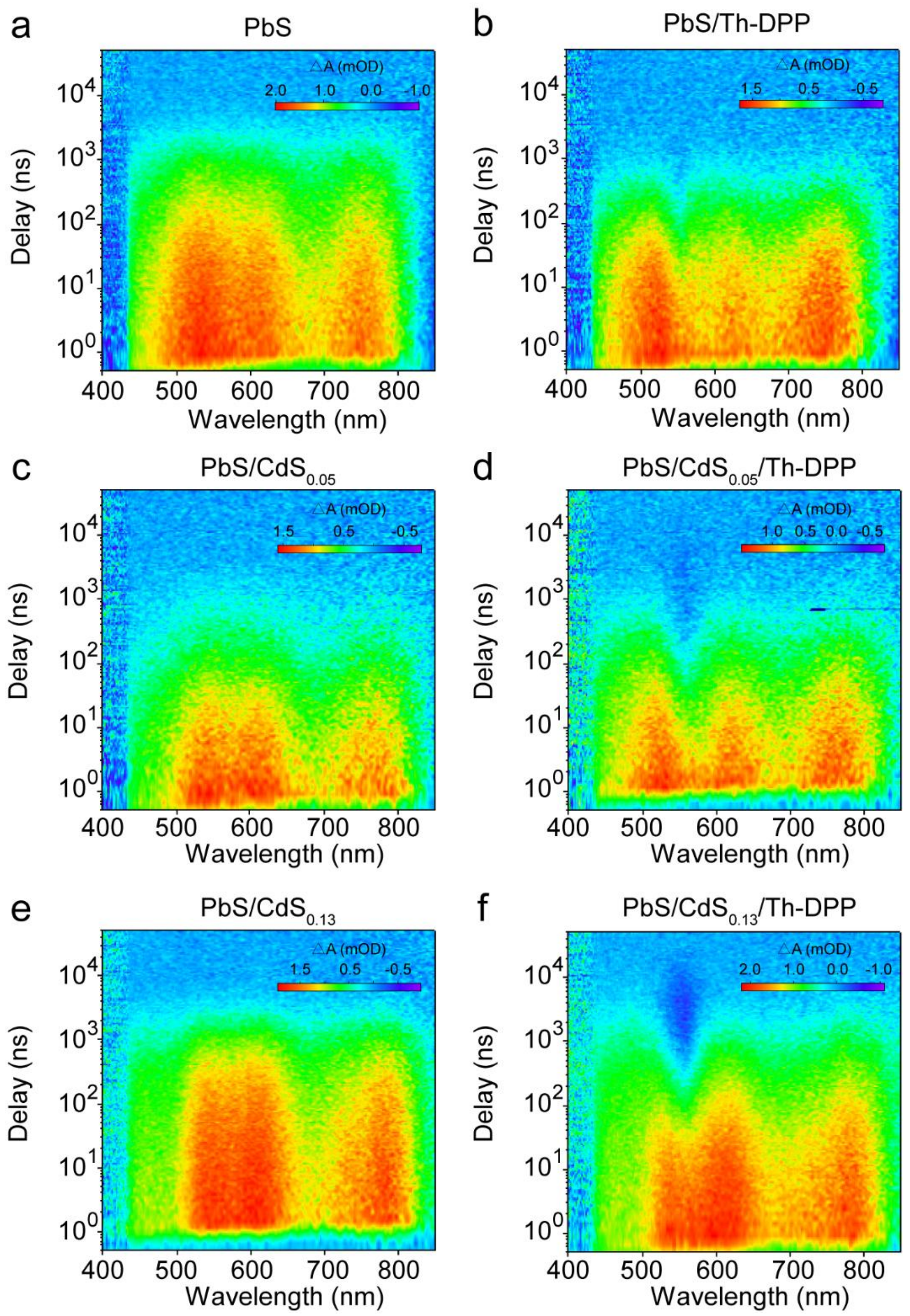

Fig. S6. Two-dimensional pseudo-colour nanosecond transient absorption spectroscopy of QDs and QDs/Th-DPP within 50 μs following 1030 nm excitation.

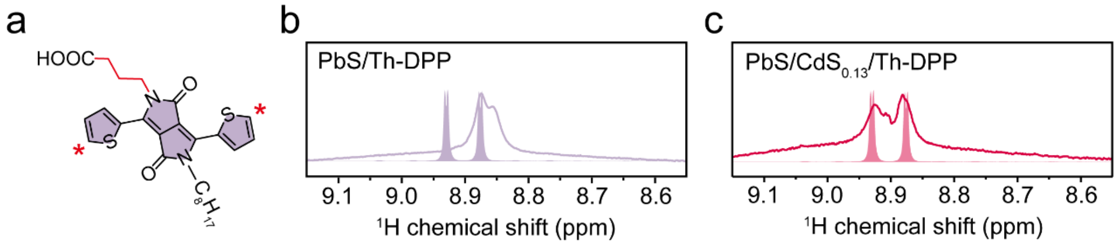

Fig. S7. (a) The molecular structure of Th-DPP, with the red asterisk indicating the proton signal studied. ^{1}H NMR spectra of Th-DPP on the surface of (b) PbS QDs and (c) PbS/CdS$_{0.13}$, in $CDCl_3$ and calibrated at 7.26 ppm. The fill line represents free Th-DPP.

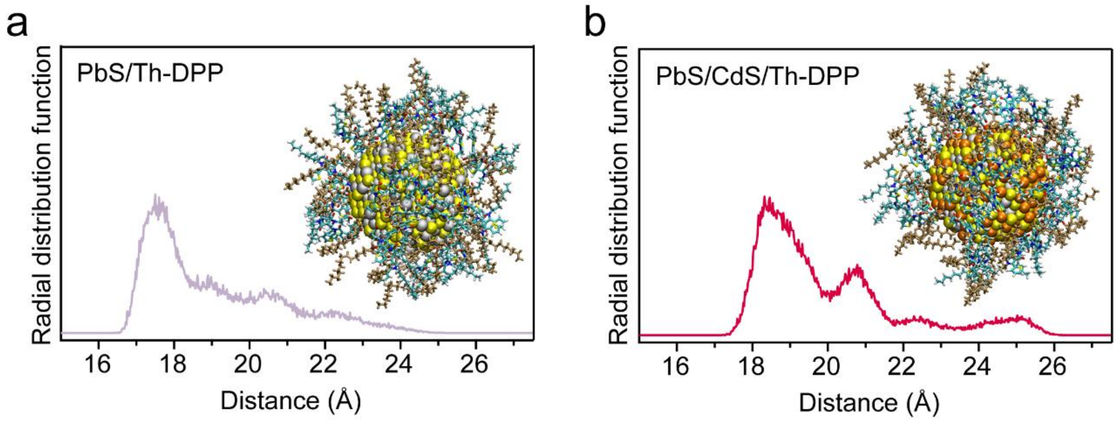

Fig. S8. Radial distribution function of (a) PbS/Th-DPP and (b) PbS/CdS/Th-DPP between the thiophene center adjacent to the anchoring group and the PbS QDs center. The insets are results of the molecular dynamics simulation.

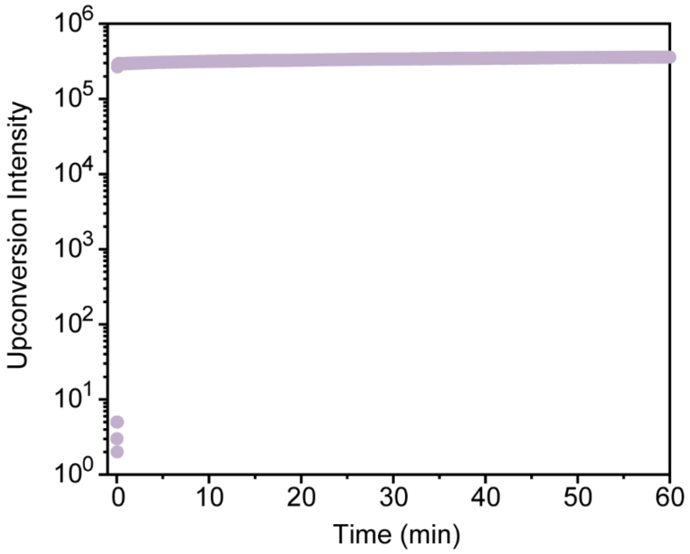

Fig. S9. Photostability of PbS/CdS$_{0.05}$/Th-DPP/rubrene under continuous 1064 nm laser (15 W/cm^2) illumination for 60 minutes in argon-saturated toluene.

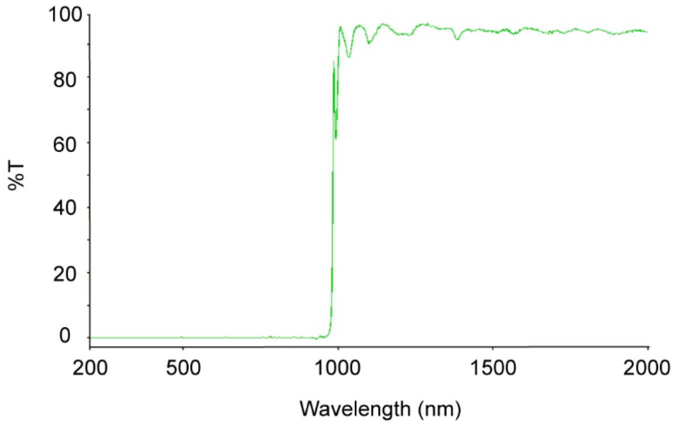

Fig. S10. The curve of the longpass filter.

Photocatalyst	Time	Volume	Results
PbS/$CdS_{0.05}$/Th-DPP rubrene	5 min	0.2 mL	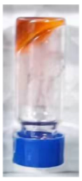
rubrene	5 min	0.2 mL	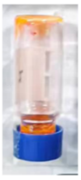

Fig. S11. The summary table of the experimental conditions and outcomes of free radical polymerization driven by beyond 1000 nm low-energy sunlight.

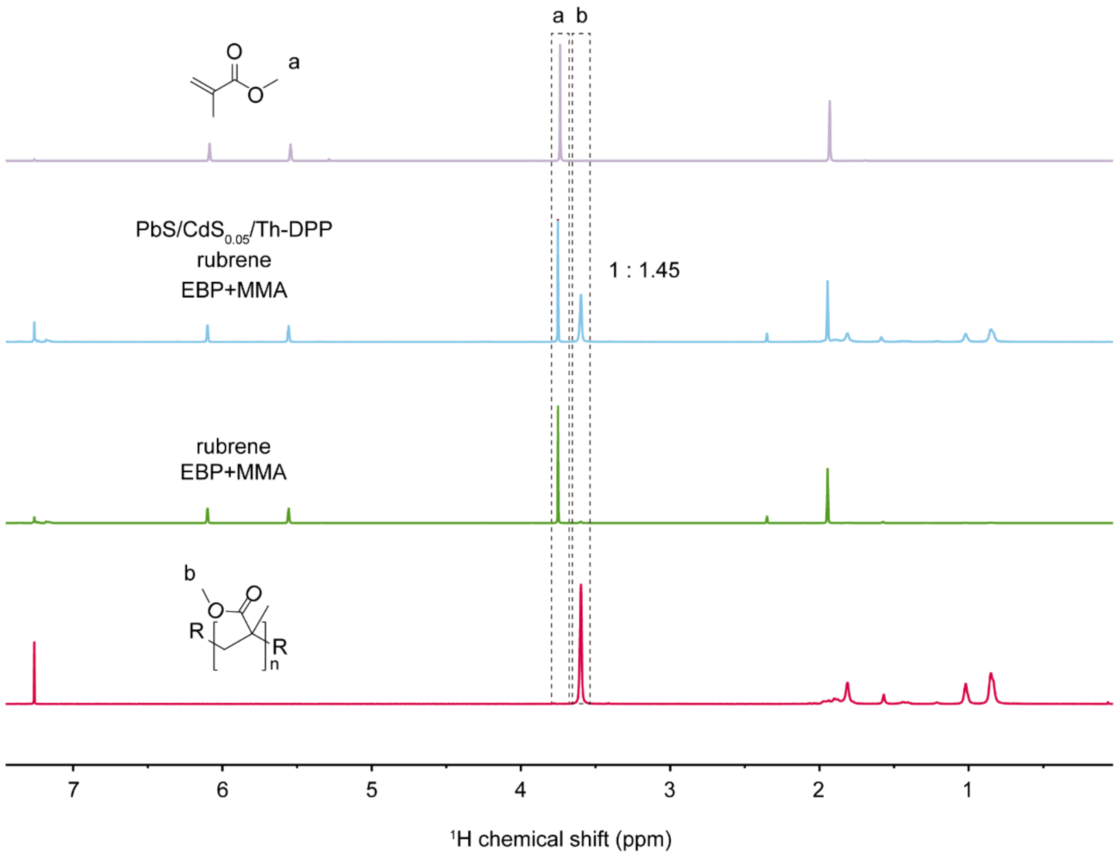

Fig. S12. ^{1}H-NMR spectrum of monomers, polymers and reaction systems after sunlight illumination in $CDCl_3$. Conversion was determined by integral following relationship, $\alpha = I_b/(I_a+I_b)$.

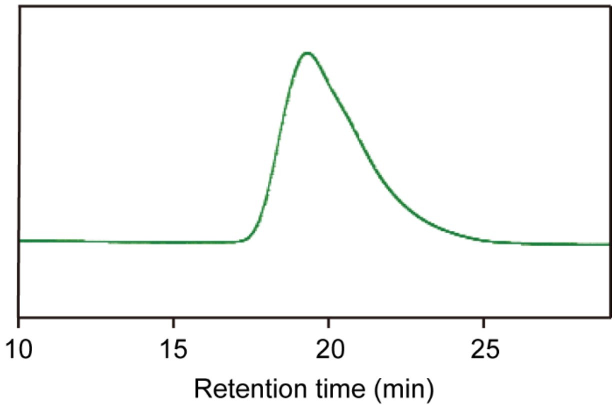

Fig. S13. GPC traces of the synthesized polymers.

4. Supporting References

1. Mahboub M, Maghsoudiganjeh H, Pham AM *et al.* Triplet energy transfer from PbS(Se) nanocrystals to rubrene: the relationship between the upconversion quantum yield and Size. *Adv Funct Mater* 2016; **26**: 6091-7.
2. Moreels I, Lambert K, Smeets D *et al.* Size-dependent optical properties of colloidal PbS quantum dots. *ACS Nano* 2009; **3**: 3023-30.
3. Huang Z, Xu Z, Mahboub M *et al.* Enhanced near-infrared-to-visible upconversion by synthetic control of PbS nanocrystal triplet photosensitizers. *J Am Chem Soc* 2019; **141**: 9769-72.
4. Lai R, Liu Y, Luo X *et al.* Shallow distance-dependent triplet energy migration mediated by endothermic charge-transfer. *Nat Commun* 2021; **12**: 1532.
5. Xu Z, Huang Z, Li C *et al.* Tuning the quantum dot (QD)/mediator interface for optimal efficiency of QD-sensitized near-infrared-to-visible photon upconversion systems. *ACS Appl Mater Interfaces* 2020; **12**: 36558-67.
6. Hess B, Kutzner C, van der Spoel D *et al.* GROMACS 4: Algorithms for highly efficient, load-balanced, and scalable molecular simulation. *J Chem Theory Comput* 2008; **4**: 435-47.
7. Essmann U, Perera L, Berkowitz ML *et al.* A smooth particle mesh Ewald method. *J Chem Phys* 1995; **103**: 8577-93.
8. Hess B, Bekker H, Berendsen HJC *et al.* LINCS: A linear constraint solver for molecular simulations. *J Comput Chem* 1997; **18**: 1463-72.
9. Ahn T-S, Al-Kaysi RO, Müller AM *et al.* Self-absorption correction for solid-state photoluminescence quantum yields obtained from integrating sphere measurements. *Rev Sci Instrum* 2007; **78**: 086105.
10. Wu W, Guo H, Wu W *et al.* Organic triplet sensitizer library derived from a single chromophore (BODIPY) with long-lived triplet excited state for triplet–triplet annihilation based upconversion. *J Org Chem* 2011; **76**: 7056-64.
11. Liang W, Nie C, Du J *et al.* Near-infrared photon upconversion and solar synthesis using lead-free nanocrystals. *Nat Photonics* 2023; **17**: 346-53.
12. Ravetz BD, Pun AB, Churchill EM *et al.* Photoredox catalysis using infrared light via triplet fusion upconversion. *Nature* 2019; **565**: 343-6.
